# Short-Term Memory Characteristics of IGZO-Based Three-Terminal Devices

**DOI:** 10.3390/ma16031249

**Published:** 2023-02-01

**Authors:** Juyeong Pyo, Jong-Ho Bae, Sungjun Kim, Seongjae Cho

**Affiliations:** 1 Division of Electronics and Electrical Engineering, Dongguk University, Seoul 04620, Republic of Korea; 2 School of Electrical Engineering, Kookmin University, Seoul 02707, Republic of Korea; 3 Department of Electronics Engineering, Gachon University, Seongnam 13120, Republic of Korea

**Keywords:** neuromorphic system, IGZO, three-terminal device, synaptic device, short-term memory

## Abstract

A three-terminal synaptic transistor enables more accurate controllability over the conductance compared with traditional two-terminal synaptic devices for the synaptic devices in hardware-oriented neuromorphic systems. In this work, we fabricated IGZO-based three-terminal devices comprising HfAlO_x_ and CeO_x_ layers to demonstrate the synaptic operations. The chemical compositions and thicknesses of the devices were verified by transmission electron microscopy and energy dispersive spectroscopy in cooperation. The excitatory post-synaptic current (EPSC), paired-pulse facilitation (PPF), short-term potentiation (STP), and short-term depression (STD) of the synaptic devices were realized for the short-term memory behaviors. The IGZO-based three-terminal synaptic transistor could thus be controlled appropriately by the amplitude, width, and interval time of the pulses for implementing the neuromorphic systems.

## 1. Introduction

The capability to store and process data is a crucial feature for handling large quantities of data without loss in the current era of big data [1]. To fulfill such demands, computing systems should be equipped with high-performance transistors. The high-performance transistors are closely related to their scalability and numbers. Assuming that the total number of transistors that can be integrated within a limited area under Moore’s law doubles every 18 to 24 months, the present capacity for transistors is tens of millions [2,3,4]. The performances and circuit functionalities can be improved by increasing the scalability and the number of transistors; however, it is difficult to increase them due to the integration limit and the heat generated during operation [5]. The von Neumann architecture is the most widely used scheme in computing systems and has an additional process of transmitting data to the memory after processor operation [6,7,8,9]. This could cause bottlenecks in data transmission [10,11]. Therefore, new architectures are being developed and evaluated in recent times instead of the serial von Neumann architecture.

Parallel structures of neuromorphic systems are currently emerging to handle large amount of data effectively [12,13]. These neuromorphic systems mimic the neurons and synapses which are connected in parallel in the biological neural networks [14]. The interconnected neurons process large quantities of data more efficiently than transistors because the neurons operate simultaneously [15,16]. If human information processing capabilities could be carried out with computing systems, energy could be consumed more efficiently for data processing [17,18,19,20].

The working of the neurons and synapses in a neuromorphic system should be duplicated concerning those of biological systems for efficient implementation. The synapses update the weights by reacting to the signals from other neurons [21]. This behavior has a large similarity with that of a memory device, but unlike existing memory systems, the results are not simply classified into “0” or “1” state [22,23]. The hardware shows several states, rather than the two on/off states. Next-generation memories such as phase-change memory (PRAM) [24], ferroelectric random-access memory (FRAM) [25], and resistive-switching random-access memory (RRAM) can be used in the neuromorphic systems [26,27]. The PRAM stores data using a material that changes into an amorphous or crystalline state by temperature. The amorphous condition is a high-resistance state (HRS), whereas the crystalline condition is a low-resistance state (LRS), which allows data to be written to and erased from a device. The HRS and LRS allow capability of realizing multiple states via varying the operation schemes. The FRAM undergoes state change by the polarization of a ferroelectric material. The RRAM stores data by the resistive-change switching of an insulator using an external electric field, where the switching characteristics are determined by the insulator and electrodes. If a reset voltage is applied, the device is switched to the HRS; conversely, if a set voltage is applied, the device is moved to the LRS. Energy efficiency for switching and the compatibility to CMOS integration are, hence, essential for high-density memory and neuromorphic systems [28,29,30,31,32].

Compared with PRAM and FRAM, the metal–insulator–metal (MIM) structure of the RRAM enables flexible fabrication and fewer material restrictions [33]. Owing to these advantages, numerous studies have been reported on RRAMs [34,35,36,37,38]. Depending on switching layer and electrode materials, the RRAM shows various switching characteristics, including unipolar [39], bipolar [35], threshold [40], long-term [41], and short-term [38], which facilitate the construction of more hardware-oriented artificial neural networks (ANNs). The three-terminal synaptic transistor that is similar to the RRAM structure was developed to enhance the integration and to allow learning and signal transmission simultaneously for advanced ANNs [42,43,44,45].

The three-terminal synaptic transistor is derived from the MOSFET structure. The interface charge traps in the channel layer are controlled by the gate voltage to achieve multistate operation capability. The two-terminal device does not learn simultaneously while receiving signals. As a limitation of the terminal, there is no distinction between the presynapse and postsynapse [46]; hence, at least three terminals (gate: presynapse, drain: postsynapse, and source: ground) are required for separating the terminals. When a signal affects the channel by the gate (presynapse), the channel conductance is altered; this activates the device to update and read data by a drain (postsynapse), which imitates the biological system. It is, therefore, possible to perform an inference operation in a learning operation concurrently so that there is less loss than in a two-terminal device [47,48].

An electrical synaptic device conducts similarly to a synapse in the biological neural network. The biological synaptic weight is the conductance from the perspective of the synaptic device. The stimulus (signal) transmitted from the presynapse (top electrode) is accumulated over time, and the increased conductance (fired weight) indicates the excitatory postsynaptic current (EPSC) that performs another signal transmission [49]. The EPSC is further triggered as an applied electrical stimulus to the gate. In another case, paired-pulse facilitation (PPF) is generated with different interval times between two pulses [50]. This means that the conductance increases by repeated pulses before recovering to the initial state, such as the process that a spike is observed before a neuron fires and returns to the steady state. Further, if the conductance is enhanced continuously by multiple pulses, it is considered as potentiation, and the opposite as depression [51]. Through the process of conductance modulation, the device realizes learning and memory operations similar to the flexible interconnects in the neural network.

In the present work, a three-terminal device was fabricated with the W gate/drain/source, HfAlO_x_ and CeO_x_ layers as gate oxides, and the IGZO channel. These materials are not only compatible with CMOS circuits but are also widely used in RRAMs. CeO_2_ is a naturally synthesized material with a single Ce^4+^ ion and two O^2−^ ions, but cerium oxide deposited by sputtering has an amorphous formation [52]. The sputtering deposited CeO_2−x_ has numerous oxygen vacancies (V_o_) within the material layer. Many V_o_ are moved by the electrical force to switch the state of the channel [53,54,55]. However, an excess of vacancies can eventually cause a leakage current from the gate to the source. This is not appropriate for neuromorphic systems requiring low power consumption. Therefore, a high-κ material is introduced to prevent unnecessary energy consumption [56]. The notable high-κ materials include HfO_2_ (gate dielectric) and Al_2_O_3_ (blocking oxide dielectric) [57]. HfO_2_ has a very high dielectric constant but weak endurance against switching. Al_2_O_3_ tends to have high endurance despite a relatively low permittivity. HfAlO_x_ formation, in which HfO_2_ and Al_2_O_3_ are stacked alternately to utilize their respective advantages, shows high endurance and permittivity [58,59]. Accordingly, the movements of many V_o_ are suppressed by the HfAlO_x_ layer to enable a low-power three-terminal synaptic transistor. IGZO is widely used in the thin-film transistors (TFT) as a channel material with a very low off-current that is also related to the leakage current [60,61,62]. Therefore, a three-terminal device was attempted in this study as a low-power neuromorphic device by adopting IGZO as the channel layer.

## 2. Materials and Methods

**Figure 1:**
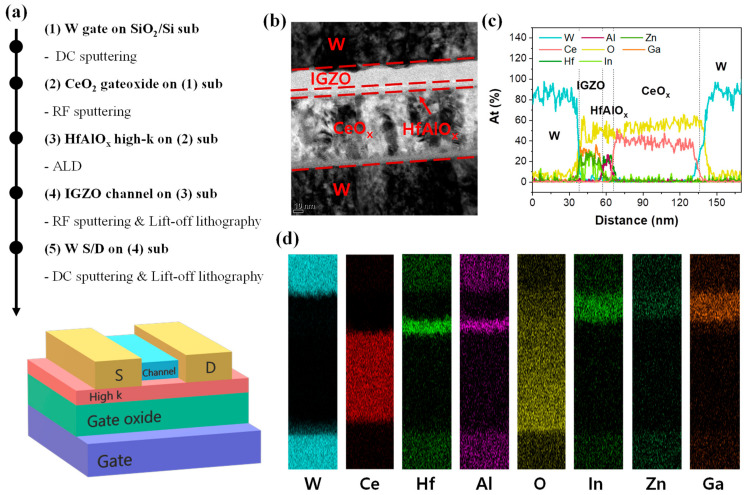
(**a**) The schematic showing 3-terminal synaptic device structure and the brief fabrication process; (**b**) HR-TEM image; (**c**) a line scanning of the atomic ratio of each element; and (**d**) an EDS mapping image. Each element exhibits colors in (**c**) and (**d**) in match; for example, the W in (**c**) and (d) is sky blue.

Figure 1a shows all the devices fabricated on SiO_2_/Si substrates. An approximately 100 nm tungsten layer was deposited as a bottom gate electrode from a commercial organization (GMEK, Anyang, Republic of Korea). A tungsten target was direct-current (DC) sputtered with Ar (20 sccm) at 3 mTorr working pressure and room temperature (RT). Then, ~30 and ~70-nm CeO_x_ layers with many oxygen vacancies were prepared on the W gate by a radio frequency (RF) magnetron sputtering system. RF sputtering is a suitable method for the reaction of the cerium target with oxygen plasma at RT. At this time, the chamber was filled with Ar (20 sccm) and O_2_ (6 sccm) while maintaining a working pressure of 5 mTorr. The CeO_x_ layers with different thicknesses only have different deposition times. Next, an ~7-nm HfAlO_x_ was grown as a current-suppressing layer on CeO_x_/W using an atomic-layer deposition (ALD) system. The nano-laminated HfAlO_x_ layer, where Hf and Al are in the ratio of 1:1, was formed by cycling HfO_2_ and Al_2_O_3_. The precursor of HfO_2_ was tetrakis (dimethylamino) hafnium (TDMAHf), and the precursor of Al_2_O_3_ was trimethylaluminum (TMA). The depositions of HfO_2_ and Al_2_O_3_ were performed at the same temperature of 355 °C, with O_3_ as the oxidant. To operate the device as a three-terminal synaptic device, an IGZO target (In:Ga:Zn = 1:1:1) was sputtered on HfAlO_x_/CeO_x_/W. The Ar had a flow rate of 10 sccm, the working pressure was 3 mTorr, and the RF power was 100 W during the sputtering. In addition, a lift-off process was used to separate the cells. The amorphous IGZO film was patterned on the device, and the IGZO channel length and width were split into 100, 200, and 400 μm. Finally, a ~100-nm layer of tungsten was formed as both the source and drain electrodes to apply electrical signals to the active IGZO layer. The W also was patterned by a lift-off process using the sputtering system. The W target was sputtered at Ar (20 sccm) and RT with 3 mTorr.

The W/IGZO/HfAlO_x_/CeO_x_/W sample vertically milled with a focused ion beam (FIB) from the source to the bottom gate was analyzed by high-resolution transmission electron microscopy (HRTEM, KANC, Suwon, Republic of Korea). In addition to acquisition of the cross-sectional image, elemental mapping and line scan were conducted simultaneously with an energy dispersive spectrometer (EDS) which is an in-situ element detector installed in the transmission electron microscopy (TEM).

To assess the synaptic properties of the device, the I−V and pulse mode were measured using a Keithley 4200 SCS semiconductor parameter analyzer (Tektronix Inc., Beaverton, OR, USA) and a 4225-PMU ultrafast current voltage pulse module, respectively. All electrical results were obtained by applying a voltage bias to the drain and bottom gate, while the source was grounded. In the I−V curves, the incremental voltage step was +0.05 V, but in the pulse mode, a dramatic increase was observed from 0 V without a step.

## 3. Results and Discussion

The image of each layer of the nanodevice, from the source to the bottom electrodes, is shown in Figure 1b. The TEM image tends to have a higher/lower contrast as the element becomes heavier/lighter [63]. The W layer, source, and bottom gate exhibit high contrasts because it is the heaviest element among the components (W, Ce, Hf, Al, O, In, Zn, Ga) of the device. The order of element weights is listed as follows: W, Hf, Ce, In, Ga, Zn, Al, and O. It is confirmed from the image that the contrast differs depending on the atomic number. Line scan (Figure 1c) and elemental mapping (Figure 1d) were performed by an EDS and TEM. The elements are represented by the same colors in both figures. The line scan shows the normalized atomic percent. The IGZO, which is the channel material, has about a 1:1:1 ratio of In, Ga, and Zn, considering the ratio of the sputtering target (In:Ga:Zn = 1:1:1). In the case of HfAlO_x_, a similar atomic ratio is observed as the proportion of Hf and Al in the ALD system. A non-stoichiometric state CeO_x_ (1.3 < x < 2) film was deposited by a sputtering system.

The initial state of the device has a very high resistance, which prevents rapid current flow. Hence, the device must be subjected to a soft breakdown by adjusting the compliance current as a forming process [64]. Similarly, the three-terminal synaptic transistor also has many defects including interface traps in the CeO_x_/HfAlO_x_/IGZO layer. These defects need to be induced that the electrons are trapped to control the conductance of the IGZO channel. The negative gate voltage releases electrons from the IGZO-HfAlO_x_ interface, whereas the positive gate voltage attracts electrons from the IGZO channel. In other words, the negative (positive) bias makes the channel more n-type (p-type) and increases (decreases) the barrier height between the source/drain and IGZO channel.

**Figure 2:**
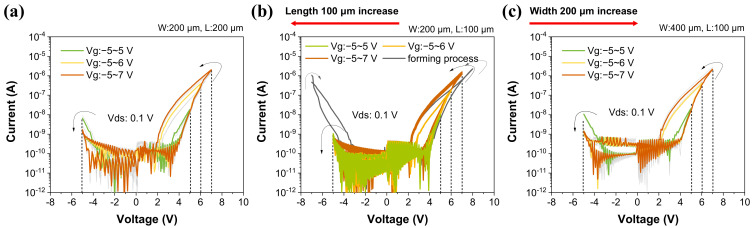
The I_d_−V_g_ curves of ~30-nm CeO_x_ with HfAlO_x_ layer device. The drain currents from (**a**) cell-to-cell measurements at W/L = 200/200 μm, (**b**) cycle-to-cycle with W/L = 200/100 μm, and (**c**) cell-to-cell with W/L = 400/100 μm at V_d_ = 0.1 V. The green, yellow, and brown lines indicate applied V_g_ in sweeping up to: 5 V, 6 V, and 7 V, respectively. The red arrows at the top indicate the changes in length and width between the measured devices in (**a**,**c**) in reference to the device in (**b**).

Figure 2 shows the I-V curves of the gate voltage versus drain current. The drain current increases as the conductance of the IGZO channel increases. In Figure 2b, the gray line indicates the I-V curve when applying a voltage bias from −7 V to 7 V to the gate terminal for the forming process. As shown in Figure S1b, the hysteresis loops from around 3 V are different with and without the forming process. The accumulated charges are a factor that controls the synaptic characteristics of the three-terminal device [65]. The drain voltage of 0.1 V is used for reading when applying voltages of −5 V to 5 V (green lines), 6 V (orange lines), and 7 V (brown lines) with width/length (μm) of 200/100 to the ~30 nm CeO_x_ device (D30), as shown in Figure 2b. The applied negative bias (−5 V) causes the device to return to its initial state. As the gate voltage increases in the positive direction, the drain current also increases, but when the gate voltage decreases in the negative direction by the DC sweep, the drain current decreases. The difference in current in the forward and backward directions also depends on the maximum gate voltage. The values of drain current ratio above gate voltage = 4 V are as follows:

$$V_{g}=5\;V,\;I_{d,after}/I_{d,before}:7.41\;V_{g}=6\;V,\;I_{d,after}/I_{d,before}:36.3\;V_{g}=7\;V,\;I_{d,after}/I_{d,before}:49.5$$

where $V_{g}$ is the gate voltage, $I_{d,after}$ is the drain current after sweeping, and $I_{d,before}$ is the drain current before sweeping. As the voltage increases, the drain current increases. This tendency also appears in Figure 2a,c which are the results applied to the same process as Figure 2b including the forming switching which is not marked. Here, the gray lines depict the cell-to-cell variations. *I_d,after_/I_d,before_* values in Figure 2a (Figure 2c) are 4.96 (3.65), 67.5 (76.7), and 147 (450) when V_g_ values are 5, 6, and 7 V, respectively. The ~70-nm CeO_x_ device (D70) in Figure S2, which has the same fabrication process but different deposition times of the CeO_x_ layer, was also measured for cycle-to-cycle and cell-to-cell (CTC) changes. Figure S2b−d shows the I-V curves during cycles and Figure S2e−g shows the I-V curves of CTC. D70 typically demonstrates that the curve shifts as charge accumulates from cycle to cycle. Even in CTC, the grain lines are curves of other cells, but they are not consistent. The D70 has the worst cycle-to-cycle and CTC variations compared to D30. The results indicate that the thick layer, which produced more V_o_, is accompanied by fluctuations [66,67]. The ~30-nm CeO_x_ device without the HfAlO_x_ layer was also fabricated for obtaining a switching material with higher dielectric constant. As the result, the drain current went significantly higher (Figure S3) than those of D30 and D70 although it was before the forming process. The sum of the current is very large if the devices used in the neuromorphic system are connected in parallel, so high-κ materials might play an essential role for low-power operation.

**Figure 3:**
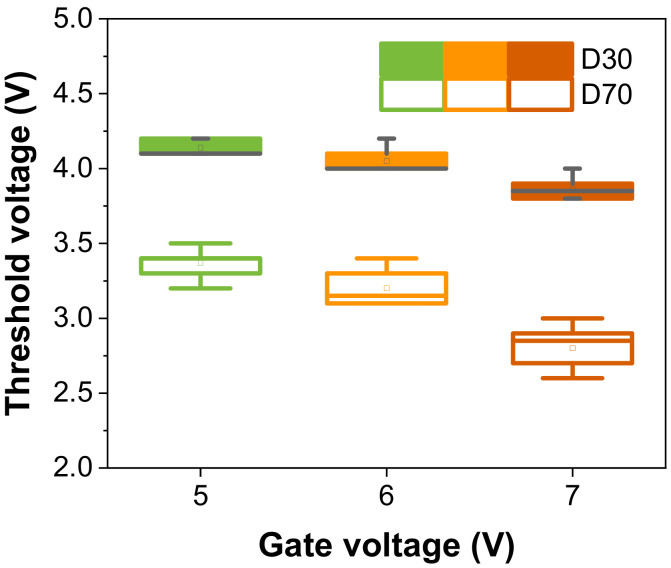
Range of fluctuation in the threshold voltage turning on the device (W/L = 200/100 μm). D30 (filled color) displays the ~30-nm CeO_x_ with HfAlO_x_ device and D70 (empty color) displays the ~70-nm with HfAlO_x_ device.

**Figure 4:**
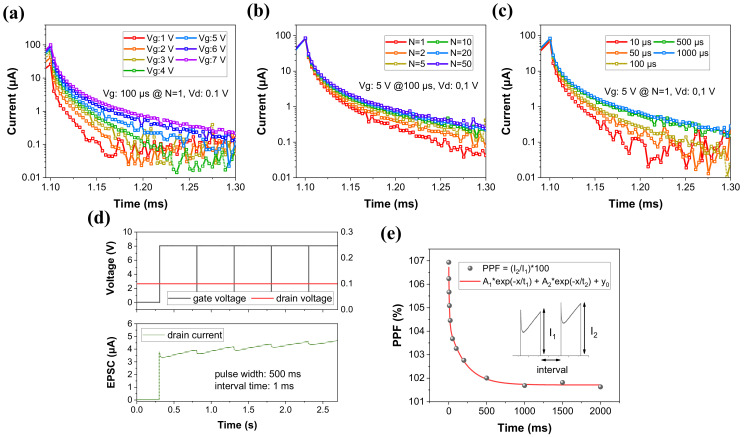
Fired currents at different (**a**) amplitudes, (**b**) pulse numbers, and (**c**) pulse widths. They are identical except for one variable and results at drain voltage of 0.1 V. (**d**) Pulse scheme (top) and EPSC (bottom). To induce EPSC, pulses of 8 V for 500 ms are repeatedly applied at an interval of 1 ms. (**e**) PPF (%); I_2_/I_1_, shows different currents increasing due to the interval time of a paired-pulse.

The threshold voltage (V_th_) is the turn-on point of the three-terminal device. The V_th_ values of D30 and D70 with 200/100 μm width/length are plotted in Figure 3. The ranges of V_th_ in D30 are 4.1–4.2 V (V_g_: 5 V), 4–4.2 V (V_g_:6 V), and 3.8–4 V (V_g_:7 V). Similarly, the ranges in D70 are 3.2–3.4 V, 3.1–3.4 V, and 2.6–3 V. The V_th_ fluctuations are relatively larger in D70 than in D30 owing to the larger V_o_. The D30 (W/L = 200/100 μm) is appropriate for a device with a small fluctuation to mimic synapses. It is an ANN system that is learned by transmitting signals derived from the stimulus to the postsynapse. For learning, the synapse is activated by updating the weights. From the device perspective, the gate acts as the presynapse (the terminal into which a pre-synaptic neuron signal comes), the drain acts as the postsynapse (the terminal through which a post-synaptic neuron signal goes out), and the channel layer acts as the weight reactant. Accordingly, it is proposed that the current of the three-terminal synaptic transistor should be changed by an electrical signal. This current is commonly referred to as EPSC. First, as shown in Figure 4a−c, an amplitude of 1–7 V (N = 1, with = 100 μs), a pulse number of N = 1–50 (amplitude = 5 V, width = 100 μs), and a pulse width of 10–1000 μs (N = 1, amplitude = 5 V) are applied to the gate terminal. Further, a bias of 0.1 V is continuously applied to the drain terminal to read the device state. The drain current increases as the amplitude, pulse number, and pulse width increase. After the pulse is removed at the gate, the triggered current gradually decreases over time, which proves the D30 device has short-term characteristics. It is also identified that the recovery time varies with the magnitude of the current generated by the pulse. Table 1 shows the decay times of the drain currents immediately after removing the pulse.

In Table 1, I_0_ is the triggered drain current directly after applying the pulse, and t_decay_ indicates the time it takes for I_0_ to reduce by 100% or more. In Figure 4d, the EPSC is obtained by applying a pulse width (interval time) of 500 ms (1 ms) and amplitude of 8 V. The resulting current tends to recover to a steady state due to the first pulse, but it accumulates as the next pulse is input. The behaviors of brain show the property of remembering for a long time if the frequency of stimulation is high, while memory duration becomes short if the frequency is low [68]. The number of frequencies can be modulated by controlling the interval time between pulses. The PPF, which measures dissimilarity between only two pulses with time, is shown in Figure 4e. The interval time is set from 1 ms to 2000 ms, and the PPF is defined as Equation (1).

(1) $$PPF\;\left(\%\right)=100\times I_{2}/I_{1}$$

In the D30 with short-term, as the interval time is longer, the difference between the first and second current is small. On the contrary, if the time is short, it has a large change of approximately 107%.

**Figure 5:**
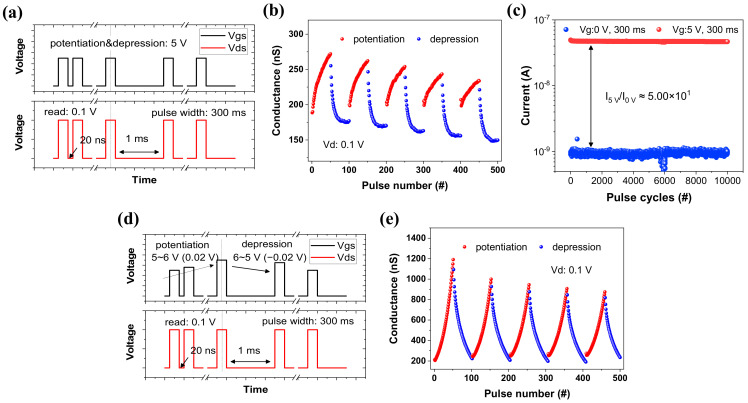
(**a**) By applying a pulse to the gate terminal (black line) and drain terminal (red line), (**b**) the conductance update of potentiation (red)/depression (blue). The pulse width is 300 ms and the amplitude is 5 V (at V_d_ = 0.1 V). In potentiation, an interval time has 20 ns, while it is 1 ms in depression. (**c**) The pulse endurance of the activated (red) and steady (blue) state during 10,000 cycles. The gate pulse is designed 5 V/300 ms for activation, and 0 V/300 ms for stabilization. (**d**) The increasing/decreasing pulse design: in potentiation and depression, 5~6 V/300 ms (step: 0.02 V) and 5~6 V/300 ms (step: −0.02 V) are applied as the input signals, respectively. (**e**) The conductance update from (**d**) gets more linear compared with that in (**b**).

Utilizing the property, short-term potentiation (STP) and short-term depression (STD) can be realized. Short-term synaptic plasticity means that synaptic efficiency changes when the period of presynapse is mirrored: STP is the increase of synaptic strength under repeated stimuli, and STD is the decrease under repeated stimuli [69]. The STP is conducted by input signals that are repeatedly applied at 5 V and 300 ms with 20 ns interval time in Figure 5b. Due to short-term characteristics, the conductance accumulated in the channel recovers over time. If the same signal is applied before returning to the steady state in time without inducing the potentiation, the reduction speed is delayed. STD takes place when the same signal with intervals of 1 ms as STP is applied to the devices. The recovering memory is delayed by controlling only the interval time of the pulse, which is the same as STP. It is possible to show the depression due to the slowdown time. The stable state is achieved when 0 V (blue) is applied to the gate terminal, and the activated state is observed when the pulse with 5 V for 300 ms (red) is applied. For 10,000 cycles, it displays extremely strong endurance and has an on/off ratio of roughly 50%. By modifying the design of the pulse, the conductance of the channel can be set as predictive. High recognition can be obtained in artificial neural simulations since the high linearity in weight update [70]. The results, as shown in Figure 5e, are obtained by applying the increasing/decreasing pulse design. The linearly-changing input scheme in potentiation or depression shows better linearity in weight change than identical-input scheme. Consequently, the D30 can control channel conductance by optimizing the pulse scheme. This is equal to the results in D70 (Figure S4a,b).

## 4. Conclusions

In conclusion, a novel three-terminal synaptic transistor have been fabricated and evaluated for application in the neuromorphic systems. The device without the HfAlO_x_ layer performs a high working current, which causes high power consumption. For the devices with thick CeO_x_ (~70 nm), the variation was worse than that with thin CeO_x_ (~30 nm). In addition, the extended channel width (200 μm to 400 μm) and length (100 μm to 400 μm) are analyzed considering the identical area to optimize the device. Experimentally, the CeO_x_ 30 nm device (D30) with HfAlO_x_ layer is appropriate as an efficient synaptic device when the channel width and length are 200 and 100 μm, respectively. In particular, the fluctuation of V_th_ was 0 to 0.1 V when the gate voltage is applied up to 5 V. The EPCS, PPF, STP, and STD have been performed to mimic the biological synapse. The larger the amplitude (7 V), the higher the pulse number (100), or the longer the pulse width (1000 μs), the higher the current. Additionally, as the current increases, the time to return to the steady state is increased, which has been proven through the PPF test. Since D30 has short-term memory characteristics, it causes potentiation or depression under different interval times between pulses. By controlling the pulse amplitude, conductance is updated linearly, making it simple to predict. Moreover, the property is a factor that allows a high recognition rate in the artificial neural network towards the online learning.

## Figures and Tables

**Table 1:** t_decay_ and I_0_ of the drain current according to amplitude, pulse number, and pulse width.

	Amplitude (V)	1	2	3	4	5	6	7
	Pulse number (N)	1	2	5	10	20	50	
	Pulse width (μs)	10	50	100	500	1000		
Figure 4a (amplitude)	t_decacy_ (ms)	0.040	0.046	0.050	0.056	0.063	0.073	0.086
	I_0_ (μA)	26.00	42.29	56.80	69.60	81.03	91.25	99.63
Figure 4b (pulse number)	t_decay_ (ms)	0.060	0.070	0.076	0.095	0.096	0.130	
	I_0_ (μA)	80.49	81.65	82.70	83.42	84.19	85.13	
Figure 4c (pulse width)	t_decay_ (ms)	0.046	0.056	0.060	0.080	0.090		
	I_0_ (μA)	70.79	78.91	80.72	82.80	83.61		

## Data Availability

Not applicable.

## Supplementary Materials

The following supporting information can be downloaded at: https://www.mdpi.com/article/10.3390/ma16031249/s1, Figure S1: Device schematic and DC characteristics; Figure S2. Dependency of current characteristics on channel dimension in the double-layer structure; Figure S3. Dependency of current characteristics on channel dimension in the single-layer structure; Figure S4. Learning operations of the ~70-nm synaptic device.
