# Supplementary material for: Short-Term Memory Characteristics of IGZO-Based Three-Terminal Devices
Source: Materials (Basel). 2023 Feb 1;16(3):1249. doi: 10.3390/ma16031249 (PMC9919079; doi:10.3390/ma16031249)
Supplement: Supplementary file 1 [file materials-16-01249-s001.zip › materials-2088255-supplementary.docx]

Supplementary Material

Short-Term Memory Characteristics of IGZO-Based
Three-Terminal Devices

Juyeong Pyo ^1^, Jong-Ho Bae ^2^ and Sungjun Kim ^1,^*, Seongjae Cho ^3,^*

| **Citation:** Pyo, J.; Bae, J.-H.; Kim, S.; Cho, S. Short-Term Memory  Characteristics of IGZO-Based Three-Terminal Devices. *Materials* **2023**, *16*, x. https://doi.org/10.3390/xxxxx  Academic Editor: Enrico Napolitani  Received: 25 November 2022  Revised: 19 January 2023  Accepted: 26 January 2023  Published: date  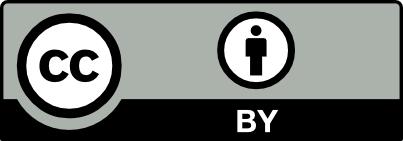  **Copyright:** © 2023 by the authors. Submitted for possible open access publication under the terms and conditions of the Creative Commons Attribution (CC BY) license (https://creativecommons.org/licenses/by/4.0/). |
| --- |

^1^ Division of Electronics and Electrical Engineering, Dongguk University, Seoul 04620, Republic of Korea;
joozero0908@gmail.com

^2^ School of Electrical Engineering, Kookmin University, Seoul 02707, Republic of Korea; jbae@kookmin.ac.kr

^3^ Department of Electronics Engineering, Gachon University,
Seongnam 13120, Republic of Korea

***** Correspondence: sungjun@dongguk.edu (S.K.), felixcho@gachon.ac.kr (S.C.)

**Figure S1.** Device schematic and DC characteristics. (a) Schematic of ~30-nm CeO_x_/HfAlO_x_ device. (b) Gate voltage versus drain current at V_d_ = 0.1 V before and after forming process. (c) Cell-to-cell drain currents after forming process from the devices with W/L=200/100 μm. The gate voltage is applied up to 5 V (green), 6 V (yellow), and 7 V (brown) reading at V_d_ = 0.1 V.

**Figure S2.** Dependency of current characteristics on channel dimension in the double-layer structure. (a) Schematic of ~70-nm CeO_x_/HfAlO_x_ device. Cycle-to-cycle measurement results for gate voltage versus drain current from the devices with (b) W/L = 200/200 μm, (c) W/L = 200/100 μm, and (d) W/L = 400/200 μm. Cell-to-cell drain currents from the devices with (e) W/L = 200/200 μm, (f) W/L = 200/100 μm, and (g) W/L = 400/200 μm. All the results have been obtained at V_d_=0.1 V. The gate voltage is applied up to 5 V (green), 6 V (yellow or orange), and 7 V (brown).

**Figure S3.** Dependency of current characteristics on channel dimension in the single-layer structure (a) Schematic of ~30-nm CeO_x_ device without HfAlO_x_. The cell-to-cell drain currents versus gate voltage from the devices with (b) W/L = 200/200 μm, (c) W/L = 200/100 μm, and (d) W/L = 400/200 μm at V_d_ = 0.1 V. The gate voltage is applied up to 5 V (green), 6 V(yellow), and 7 V (brown).

**Figure S4.** Learning operations of the ~70-nm CeO*x* synaptic device. Potentiation (red) and depression (blue) preceded by (a) identical pulses and (b) linearly increasing/decreasing pulses. (c) Identical pulses: amplitude of 5 V and pulse width of 100 ms. (d) Linearly ncreasing/decreasing pulses with amplitude of 5~6 V (step: 0.02 V) and pulse width of 100 ms. The drain pulse (red line) is applied at the same time with gate pulse (black line).
